# Organismal Protein Homeostasis Mechanisms

**DOI:** 10.1534/genetics.120.301283

**Published:** 2020-06-24

**Authors:** Thorsten Hoppe, Ehud Cohen

**Affiliations:** * Institute for Genetics and Cologne Excellence Cluster on Cellular Stress Responses in Aging-Associated Diseases (CECAD) and; † Center for Molecular Medicine Cologne (CMMC), University of Cologne, Cologne 50931, Germany and; ‡ Department of Biochemistry and Molecular Biology, the Institute for Medical Research Israel-Canada (IMRIC), the Hebrew University School of Medicine, Jerusalem 91120, Israel; § Center for Molecular Medicine Cologne (CMMC), University of Cologne, Cologne 50931, Germany and Department of Biochemistry and Molecular Biology, the Institute for Medical Research Israel-Canada (IMRIC), the Hebrew University School of Medicine, Jerusalem 91120, Israel

**Keywords:** *C. elegans*, proteostasis, proteotoxicity, proteasome, ubiquitin, chaperone, autophagy, stress response, unfolded protein response, intertissue signaling, WormBook

## Abstract

Sustaining a healthy proteome is a lifelong challenge for each individual cell of an organism. However, protein homeostasis or proteostasis is constantly jeopardized since damaged proteins accumulate under proteotoxic stress that originates from ever-changing metabolic, environmental, and pathological conditions. Proteostasis is achieved via a conserved network of quality control pathways that orchestrate the biogenesis of correctly folded proteins, prevent proteins from misfolding, and remove potentially harmful proteins by selective degradation. Nevertheless, the proteostasis network has a limited capacity and its collapse deteriorates cellular functionality and organismal viability, causing metabolic, oncological, or neurodegenerative disorders. While cell-autonomous quality control mechanisms have been described intensely, recent work on *Caenorhabditis elegans* has demonstrated the systemic coordination of proteostasis between distinct tissues of an organism. These findings indicate the existence of intricately balanced proteostasis networks important for integration and maintenance of the organismal proteome, opening a new door to define novel therapeutic targets for protein aggregation diseases. Here, we provide an overview of individual protein quality control pathways and the systemic coordination between central proteostatic nodes. We further provide insights into the dynamic regulation of cellular and organismal proteostasis mechanisms that integrate environmental and metabolic changes. The use of *C. elegans* as a model has pioneered our understanding of conserved quality control mechanisms important to safeguard the organismal proteome in health and disease.

Sustaining a healthy proteome is a lifelong challenge of an organism. Proteostasis is achieved via a network of quality control pathways that orchestrate protein folding and degradation. The proteostasis network (PN) has a limited capacity and its collapse deteriorates organismal viability. Recent work in *Caenorhabditis elegans* has demonstrated the systemic coordination of proteostasis between distinct tissues of an organism. These findings indicate the existence of intricately balanced PNs important for the integration and maintenance of the organismal proteome. Here, we describe the coordination of protein quality control pathways with an emphasis on *C. elegans* studies that have pioneered the understanding of quality control mechanisms important for health and disease.

## The PN

**Figure 1:**
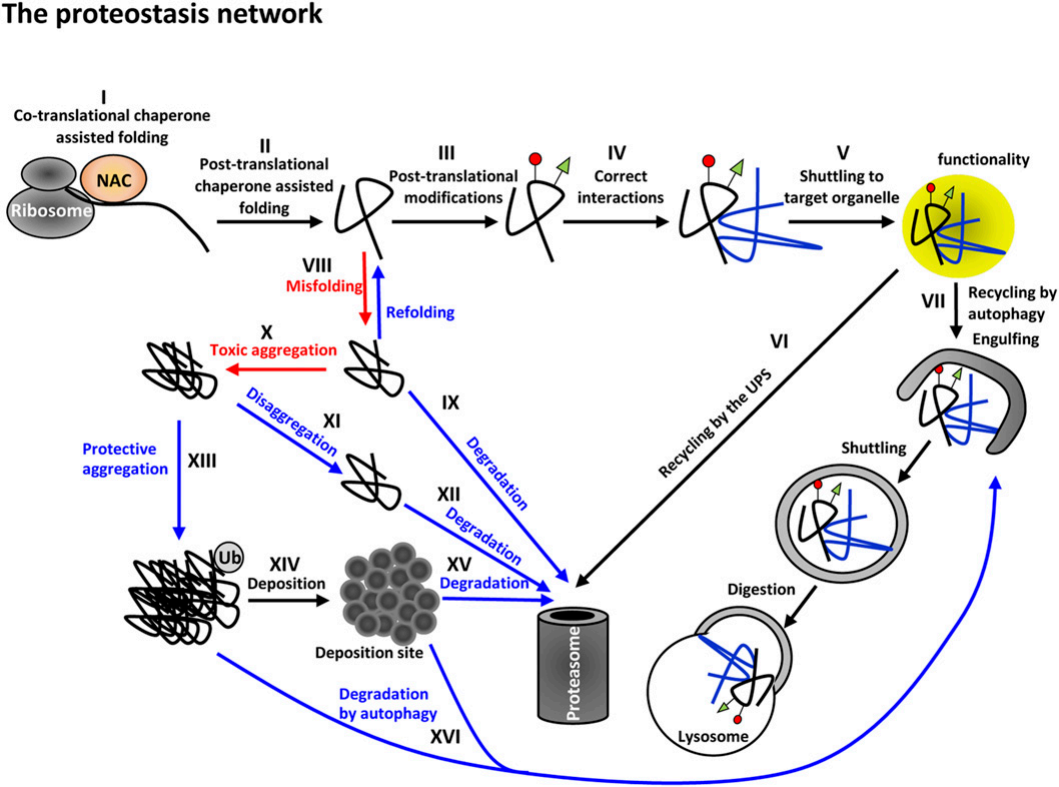
The proteostasis network (PN). The folding of nascent polypeptides is cotranslationally assisted by specialized chaperones such as the nascent polypeptide-associated complex (NAC) (I). Interacting chaperones further assist the folding of the newly synthesized polypeptide (II), and additional components of the PN safeguard the integrity of post-translational modifications (III) and intermolecular interactions (IV). The fully processed protein is shuttled to its target organelle where it executes enzymatic or structural functions (V). When the mature protein is damaged or should be recycled, it undergoes degradation by the ubiquitin (Ub)/proteasome system (UPS) (VI) or autophagy (VII). Even under normal conditions a fraction of the nascent polypeptides fail to fold properly (VIII). These misfolded proteins can be refolded by specialized chaperones to attain their correct spatial structures and become functional. Molecules that fail to fold properly are degraded by the 26S proteasome (IX). Upon accumulation, aggregation-prone, misfolded proteins form toxic oligomers (X) that can be detoxified either by disaggregation (XI) and subsequent UPS-mediated degradation (XII), or by protective hyperaggregation (XIII). Under certain conditions, these aggregates are transported to cellular deposition sites (XIV), to subsequently be degraded by the 26S proteasome (XV) or by autophagy (XVI).

The proteome is defined as the complete set of proteins expressed in a given cell type or organism, which can vary with time and physiological status (Balchin *et al.* 2016). Since the integrity of the proteome is critical for cellular and organismal functionality and viability, numerous quality control pathways act together to maintain proteostasis. For example, molecular chaperones support efficient folding of nascent polypeptides synthesized at the ribosome to secure their biological function(s) (Balchin *et al.* 2016), whereas protein degradation mechanisms degrade damaged and unneeded proteins (Kwon and Ciechanover 2017). In contrast to nascent, metastable polypeptides, which can adopt numerous alternative formations, fully folded proteins attain three-dimensional structure(s) that allow correct functionality and proper binding to interaction partners. Accordingly, the predetermined spatial conformation of a protein has to be achieved during or shortly after translation, and is maintained throughout its entire lifetime. To support the integrity and functionality of the proteome, eukaryotic cells have evolved a nexus of sophisticated molecular mechanisms, summarized as the PN (Balch *et al.* 2008) (Figure 1). The PN supervises the integration and balance of intermolecular protein interactions with cellular transport and/or signaling pathways. The coordination between the different proteostatic nodes is tightly adjusted in response to proteotoxic stress caused by environmental and metabolic challenges (Powers *et al.* 2009; Noormohammadi *et al.* 2018). The human PN involves > 1000 accessory factors and regulatory components, which govern protein synthesis, folding, and degradation (Powers *et al.* 2009). Defects in the folding of nascent polypeptides, and in the refolding or elimination of damaged proteins, ultimately result in an accumulation of toxic protein aggregates, which endanger the integrity of the entire proteome (Balchin *et al.* 2016). Cotranslational processing of newly synthesized proteins is fundamental for efficient protein folding. Protein biogenesis is supported by recognition of the nascent polypeptide chain already inside the ribosomal tunnel (Gamerdinger *et al.* 2019). This tunnel-discriminating activity is provided by the nascent polypeptide-associated complex (NAC) at the ribosomal exit site where it teams up with additional molecular chaperones to ensure correct folding of newly translated polypeptides (Kirstein-Miles *et al.* 2013). Recent work in *C. elegans* has demonstrated that NAC is critical for organismal viability and import of proteins into the endoplasmic reticulum (ER). Similarly to NAC, the ribosome-associated complex, which is composed of chaperones including a member of the heat shock protein (HSP) 70 family, assist nascent polypeptides to attain the correct spatial structure (Deuerling *et al.* 2019). Post-translational control of protein folding is supported by HSP40 and TRiC, two specialized cytosolic chaperones (Hartl and Hayer-Hartl 2002). Secretory proteins are processed and folded upon transport into the ER lumen. The majority of these proteins contain an N-terminal ER localization signal, which is cleaved off by the signal peptidase (Haeuptle *et al.* 1989). Subsequent folding of the inserted polypeptides is supported by N-linked glycosylation and ER-resident quality control chaperones including calnexin, calreticulin, the protein disulfide-isomerase, and cyclophilin B (Ou *et al.* 1993; Meunier *et al.* 2002; Shiraishi *et al.* 2011; Dubnikov *et al.* 2016, 2017). Accordingly, increased synthesis of N-glycan precursors or N-acetylglucosamine supplementation induces distinct protein quality control mechanisms, and extends life span in *C. elegans* via modulation of the ER-linked integrated stress response (ISR) (Denzel *et al.* 2014; Horn *et al.* 2020).

In case protein refolding cannot be sufficiently executed, chaperones team up with the ubiquitin/proteasome system (UPS) or the autophagy–lysosome pathway to trigger degradation of misfolded proteins (Kettern *et al.* 2010; Ulbricht and Hohfeld 2013; Vilchez *et al.* 2014; Kevei *et al.* 2017; Tawo *et al.* 2017) (Figure 1). The UPS is one major proteolytic component of the cellular PN regulating the degradation of regulatory or damaged proteins (Vilchez *et al.* 2014; Dikic 2017). Turnover by the 26S proteasome is highly selective and initiated by covalent attachment of the small, evolutionarily conserved protein ubiquitin, predominantly to internal lysine residues (Marques *et al.* 2009). Substrate ubiquitylation is a dynamic process mediated by an enzymatic cascade that involves ubiquitin-activating enzymes (E1), ubiquitin-conjugating enzymes (E2), and ubiquitin protein ligases (E3). This process is tightly regulated and could be reversed by deubiquitylation enzymes.The autophagy–lysosome pathway is the other main proteolytic system that supports proteostasis and cellular recycling processes (Meléndez and Levine 2009) by turning over damaged and aggregated proteins, protein complexes, and even whole organelles (Vilchez *et al.* 2014; Khaminets *et al.* 2016; Dikic 2017). A characteristic hallmark of autophagy is the formation of double-membrane autophagosomes, which engulf their particular cargo substrate and deliver it to the lysosome for degradation. Substrate selectivity is provided by specific cargo–ligand–receptor recognition and usually involves ATG8/LC3, which couples the cargo directly with the autophagy apparatus. Despite the differences in substrate selection and turnover rates of each pathway, the UPS and autophagy share mechanistic aspects, and signaling concepts of ubiquitin attachment, recognition, and hydrolysis (Kirkin *et al.* 2009; Wong and Cuervo 2010; Khaminets *et al.* 2016; Liebl and Hoppe 2016; Dikic 2017). The intricate coordination between protein folding and degradation is either connected by direct interaction between chaperones and specialized E3 ubiquitin ligases (chaperone-assisted ubiquitylation), or by chaperone-mediated cargo uptake at the lysosomal or endosomal membrane (chaperone-mediated autophagy) (Kettern *et al.* 2010).Keeping protein biogenesis and degradation in proper balance is challenging, especially with regard to environmental stress conditions, with aging or inherited disease-associated mutations (Hartl *et al.* 2011). Most neurodegenerative disorders are associated with collapse of the PN ultimately caused by the accumulation of aberrantly aggregated polypeptides that are not correctly folded, and that have escaped quality control and degradation. This disastrous failure of proteostasis is modulated by external risk factors or disease-related mutations, suggesting a limited capacity of the PN. In line with this idea, increased activity of certain quality control pathways could postpone protein aggregation and disease onset in various model organisms (Taylor and Dillin 2011; Vilchez *et al.* 2014; Labbadia and Morimoto 2015). For instance, overexpression of proteasomal subunits increases stress resistance and degradation of misfolded proteins in worms due to enhanced activity of the 26S proteasome (Tonoki *et al.* 2009; Vilchez *et al.* 2012).

## Coordination of PNs by Organelle-Specific Specialized Stress-Response Programs

**Figure 2:**
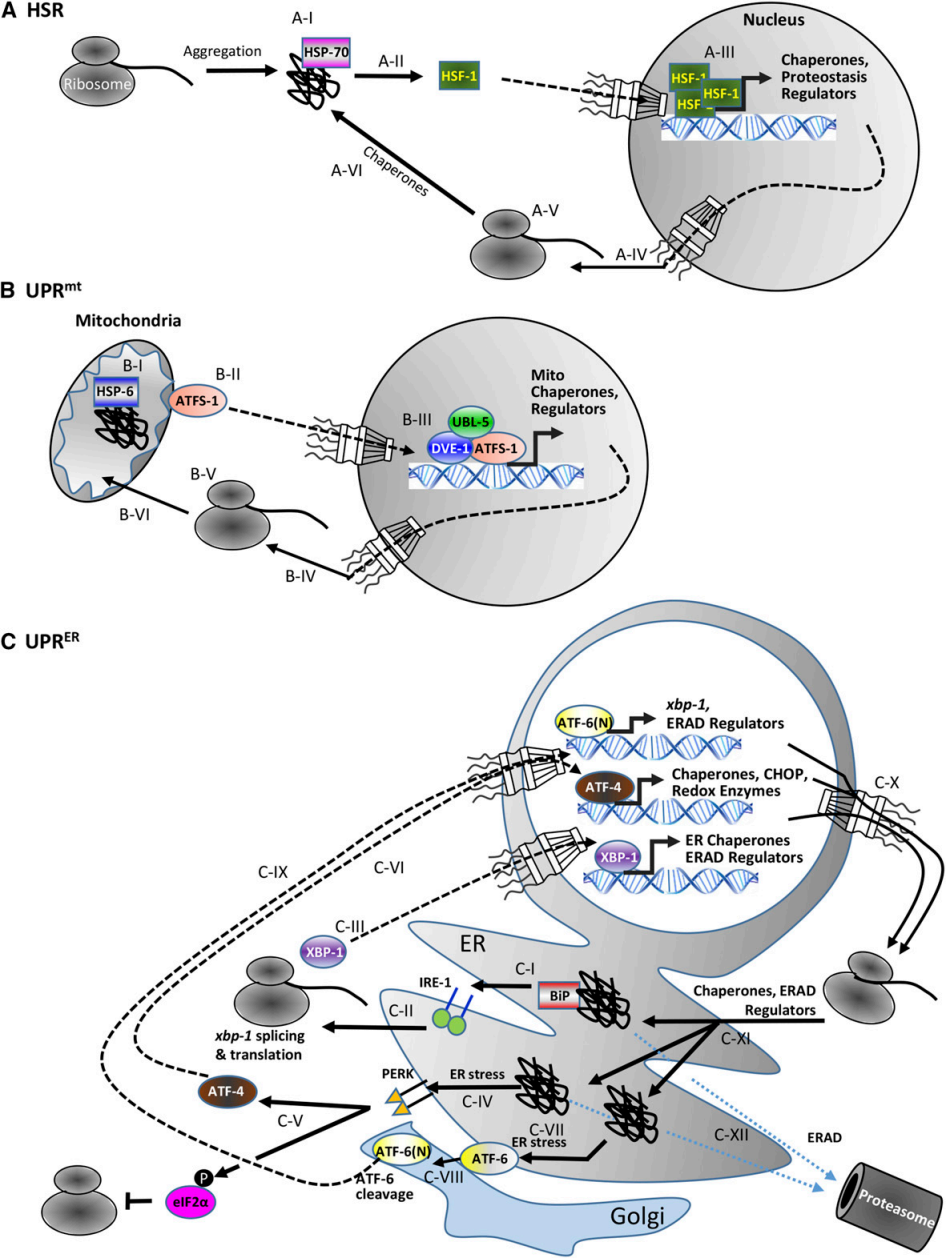
Stress-response mechanisms. The accumulation of protein aggregates in different cellular organelles activates organelle-specific stress-response mechanisms that modulate gene expression to support the restoration of proteostasis. (A) Heat exposure leads to the accumulation of aggregated proteins within the cytosol and to the activation of the heat-shock response (HSR). A cytosolic chaperone, a member of the HSP70 family, recognizes protein aggregates (A-I) and mediates the translocation of the transcription factor HSF-1 into the nucleus (A-II), where it trimerizes and regulates the expression of its target genes (A-III). The resulting transcripts migrate to the cytosol (A-IV) and the translated proteins, mostly chaperones (A-V), support proteostasis maintenance (A-VI). (B) A similar mechanism, known as the mitochondrial unfolded protein response (UPR^mt^), is caused by metabolic impairment such as oxidative stress or malfunction of the electron transport chain in mitochondria. Increased levels of protein aggregates are detected by the HSP70 chaperone, HSP-6 (B-I), which induces the migration of the transcription factor ATFS-1 into the nucleus (B-II). In the nucleus, ATFS-1 teams up with UBL-5 and the chromatin modulator DVE-1 to change the expression levels of its target genes (B-III). The resulting messenger RNA transcripts are exported into the cytosol (B-IV) where they are translated (B-V) and transported into mitochondria to restore proteostasis. (C) The ER has three canonical unfolded protein response mechanisms (UPR^ER^) that are activated upon accumulation of aggregated proteins within the ER lumen. Similarly to the HSR and the UPR^mt^, a member of the HSP70 family named BiP identifies misfolded proteins and activates IRE-1 (C-I), which initiates a process of splicing and translation of the transcription factor XBP-1 (C-II). XBP-1 migrates to the nucleus (C-III) where it activates the expression of genes that encode for ER chaperones and components of the ER-associated protein degradation (ERAD) pathway. The protein aggregation-mediated ER stress further activates the kinase PERK, which has two key functions (C-V): it promotes the migration of ATF-4 into the nucleus (C-VI) where it regulates its target gene networks and phosphorylates eIF2α. The phosphorylation of eIF2α inhibits the translation of proteins that require the assistance of chaperones to fold properly, thereby easing the workload of the ER-resident proteostasis network. The third UPR^ER^ mechanism that is activated upon ER stress involves ER membrane-bound ATF-6 (C-VII), which is shuttled to the Golgi apparatus, where it is proteolytically cleaved (C-VIII) enabling shuttling into the nucleus (C-IX). In the nucleus, it enhances the expression of *xbp-1* and additional target genes encoding ERAD components. The transcripts that are formed as a result of the activities of XBP-1, ATF-4, and ATF-6(N) are exported to the cytosol (C-X) and translated by the ribosome. The resulting ER-resident chaperones as well as ERAD components are transported into the ER to maintain proteostasis, either by refolding of aggregated proteins (C-XI) or proteasomal degradation of terminally misfolded proteins (C-XII).

Organisms are often exposed to unfavorable conditions that vitiate their cellular and interorgan functions. To enhance chances of survival and cope with these environmental insults, different organelle-specific stress-response mechanisms have been developed. Under proteotoxic conditions such as elevated temperature, the induction of different quality control pathways is coordinated by conserved stress-response programs (Lindquist 1986; Morimoto 1998). One major branch of the molecular machinery triggers the expression of molecular chaperones in the cytosol. These proteins are collectively defined as HSPs and the underlying induction mechanism has been termed the heat-shock response (HSR) (Figure 2). Among other stressors, exposure to high temperature triggers protein misfolding and destabilization of the proteome. Increased binding of molecular chaperones of the HSP70 family to misfolded proteins results in nuclear transport, oligomerization, and activation of heat shock factor 1 (HSF-1) to drive the expression of *hsp* genes (Sarge *et al.* 1993). The upregulated HSPs work together to refold damaged proteins and restore functional proteostasis in the cytosol (Morimoto 1998). Proteostasis-promoting communication between the nucleus and the cytosol is also conferred by the Linker of Nucleoskeleton and Cytoskeleton (LINC), a protein complex that links the cytoskeleton and the nucleoskeleton (Starr and Fridolfsson 2010). The knockdown of LINC components modulates gene expression, impairs UPS-mediated protein degradation, and exposes the worm to the toxicity of aggregation-prone proteins (Levine *et al.* 2019).

Similarly, the unfolded protein responses (UPR) are specialized mechanisms that avoid protein misfolding and aggregation in the ER (UPR^ER^) and mitochondria UPR^mt^ (Senft and Ronai 2015) (Figure 2). The UPR^ER^ is triggered by an accumulation of unfolded proteins in the ER (Walter and Ron 2011; Frakes and Dillin 2017), whereas an increase of unassembled mitochondrial protein complexes elicits the UPR^mt^ (Haynes and Ron 2010; Quiros *et al.* 2016). ER stress induces four different branches of the UPR^ER^ program. Three of them are conserved mechanisms sharing common principles comparable with the HSR (Walter and Ron 2011), while the fourth is activated when these UPR^ER^ pathways are blocked (Urano *et al.* 2002). Binding of misfolded proteins to the Hsp70 homolog BiP/Grp78 within the ER lumen, elicits the UPR^ER^, which increases the transcription of genes encoding for ER resident chaperones and factors supporting ubiquitin-driven ER-associated protein degradation (ERAD). The UPR^ER^ is actually triggered by signaling pathways downstream of activating transcription factor (ATF)-6, IRE-1, and protein kinase R-like ER kinase (PERK), which ultimately activate the transcription factors ATF-6(N), X-box-binding protein (XBP)-1, and ATF-4. While all transcription factors elevate the expression of genes encoding for proteins that increase the folding capacity of the ER, PERK and IRE-1 also reduce the translation of proteins that require the assistance of ER chaperones to mature properly (Walter and Ron 2011). Activation of the ER membrane-bound stress sensor PERK triggers phosphorylation of the eukaryotic translation initiation factor 2 α (eIF2α), which affects global protein synthesis to reduce the burden on the protein folding machinery (Harding *et al.* 1999). Whereas translation of most messenger RNAs is blocked by eIF2α regulation, specific transcripts become induced, including ATF-4. In conclusion, PERK phosphorylation is a key event of the so-called ISR, which triggers an adaptive pathway boosting the PN upon acute stress conditions. Besides these canonical branches of the UPR, work by the Ron laboratory on regulation of the BiP homolog HSP-4 has identified an additional UPR^ER^ mechanism in *C. elegans*, which is specifically activated when the canonical mechanisms are blocked (Urano *et al.* 2002).

The UPR^mt^, which was first described in mammalian cells (Zhao *et al.* 2002) and later in worms (Yoneda *et al.* 2004), functions similarly to the HSR and UPR^ER^ (Figure 2). Mitochondrial proteostasis imbalance detected by the HSP70-related chaperone HSP-6 triggers mitochondria-to-nucleus communication by activating the two transcription factors ATFS-1 and DVE-1. Upon induction of UPR^mt^, ATFS-1 and DVE-1 translocate into the nucleus for the upregulation of stress-responsive genes, including mitochondrial chaperones and proteases (Nargund *et al.* 2012; Tian *et al.* 2016). Both the stability and activity of ATFS-1 and DVE-1 are governed by post-translational events in *C. elegans*. Specifically, the small ubiquitin-like modifier (SUMO) and the activity of the SUMO-specific peptidase ULP-4 regulate the UPR^mt^ by controlling the stability of ATFS-1 and modulating the cellular localization of DVE-1 (Gao *et al.* 2019). Chaperones synthesized upon UPR^mt^ activation translocate into the mitochondria and team up to restore proteostasis (Martinus *et al.* 1996; Haynes and Ron 2010). A similar stress-responsive mechanism supports mitochondrial integrity in mammals, by sensing the proteostatic status and inducible activation of the ATFS-1-related transcription factor ATF5 (Fiorese *et al.* 2016). The balanced coordination of the described transcriptional programs and quality control mechanisms is key for organismal adaptation toward environmental and metabolic changes, precluding an accumulation of misfolded and aggregated proteins.

## Proteostasis Collapse and Conformational Disease Pathology

Despite the balanced and tightly buffered character of the PN, it has a limited capacity, which is particularly challenged under chronic stress conditions. Proteostasis collapse can be triggered by extensive organismal exposure to environmental insults, pathogenic mutations, or aging, which results in an accumulation of aggregation-prone proteins and ultimate formation of protein aggregates (Dubnikov *et al.* 2017). Such protein aggregates are toxic species, which are often associated with the manifestation of devastating diseases collectively known as “proteinopathies” (Paulson 1999). Late-onset proteinopathies include neurodegenerative disorders such as Alzheimer’s disease (AD), Parkinson’s disease (PD) (Selkoe 2003), Huntington’s disease (HD) (Bates 2003), amyotrophic lateral sclerosis (ALS) (Ticozzi *et al.* 2011), limbic-predominant age-related TDP-43 encephalopathy (Nelson *et al.* 2019), prion disorders (Aguzzi and Calella 2009), and tauopathies (Götz *et al.* 2019). While all these disorders are tightly linked to proteotoxicity, the differences in onset and development are related to the aggregation properties of the underlying disease-associated proteins. AD is induced by the accumulation and aggregation of the β amyloid (Aβ) peptide, a cleavage product of the amyloid precursor protein (APP) produced by two proteases, the β- and γ-secretases. β-secretase is a single-protein β amyloid-cleaving enzyme, and the γ-secretase complex is composed of four proteins; presenilin 1 and 2 (PS1 or PS2), nicastrin, and APH (Wolfe and Selkoe 2014). The proteases PS1 and PS2 are components of the γ-secretase complex. Mutations in PS1, PS2, or in APP cause familial AD (fAD). Interestingly, many fAD-causing mutations in PS1 lead to reduced activity of this protease and lower the production of Aβ (Ben-Gedalya *et al.* 2015; Szaruga *et al.* 2015; Xia *et al.* 2015). While it is plausible that more than one mechanism leads to the development of dementia in AD patients, the formation of Aβ oligomers, rather than large fibrils (Shankar *et al.* 2008; Cohen *et al.* 2009), appears to initiate cellular processes that culminate with synaptic dysfunction and neuronal loss underlying neurodegeneration and dementia (Long and Holtzman 2019). To detoxify Aβ oligomers, the PN exhibits two opposing activities. Under favorable cellular conditions the PN promotes the disintegration of toxic oligomers and supports their degradation. However, when these highly toxic structures exceed the PN’s capacity to disaggregate, oligomers are assembled to create large fibrils of lower toxicity (Cohen *et al.* 2006). In yeast, the chaperone HSP104 promotes disaggregation when the aggregation load is below a certain threshold and hyperaggregation when the rate of aggregation is higher than the threshold (Shorter and Lindquist 2004). In *C. elegans*, the disaggregate activity is exhibited by HSP-110, which disrupts proteotoxic aggregates together with HSP-40 and a member of the HSP-70 family (Rampelt *et al.* 2012).

Polyglutamine expansion diseases constitute another group of late-onset proteinopathies, which result from abnormally long expansions of CAG repeats, coding for polyglutamine (polyQ) stretches in the causative proteins (Paulson *et al.* 2000). When longer than a certain threshold, these tracts render the proteins prone to aggregation and cause disease later in life (Labbadia and Morimoto 2013). The group of polyQ-associated disorders includes HD, caused by the aggregation of the protein huntingtin (Bates 2003), and Machado–Joseph disease (MJD) (or spinocerebellar ataxia type 3), resulting from the aggregation of the deubiquitylating enzyme ataxin-3 (Schöls *et al.* 2004). PD is typically associated with the loss of dopaminergic neurons in the substantia nigra, and with the appearance of inclusions that contain aggregates of the presynaptic protein α-synuclein (α-syn) (Lee and Trojanowski 2006). Tauopathies originate from the aggregation of hyperphosphorylated or mutated τ, a microtubule-associated protein that is predominantly expressed in the neurons. Such τ aggregates are found in AD, and frontotemporal dementia and ALS, two disorders that share key pathomechanistic aspects (Robberecht and Philips 2013). Similarly, aggregated prion protein (PrP) is accountable for the development of familial and sporadic neurodegenerative illnesses known as prion disorders (Aguzzi and Calella 2009).

## Modeling Protein Aggregation in *C. elegans*

The short lifecycle, transparency, and amenability to genetic manipulation of *C. elegans* designates it as an excellently suited model for the investigation, and characterization, of fundamental proteostasis mechanisms underlying neurodegenerative disorders and age-related diseases (Volovik *et al.* 2014a). Various worm models based on the expression of aggregation-prone, disease-causing proteins have been studied over the recent years (Li and Le 2013). A successful protein aggregation model has to recapitulate a progressive phenotype that can be followed and measured within the short lifetime of the nematode. This can be achieved by tissue-specific expression of an aggregation-prone protein. Expression of a highly toxic metastable protein under the control of a strong promoter that drives high rates of transcriptional activity will probably lead to an immediately aggressive phenotype. Otherwise, usage of a weak promoter combined with a moderately toxic aggregating protein might not produce a detectable phenotype. Given this delicate balance between transgene expression and the level of proteotoxicity, not all disease-related worm models exhibit similar phenotypes and several physiological assays have been developed to measure even small differences in toxicity (Volovik *et al.* 2014a).

An efficient, widely used model for studying the proteotoxicity of the Aβ peptide is the CL2006 strain. In these animals, the human Aβ_3-42_ (McColl *et al.* 2009) peptide is expressed under control of the muscle-specific *unc-54* promoter. It drives Aβ production in striated body-wall muscle cells (Link 1995), leading to a progressive, age-dependent paralysis within the worm population. This phenotype can be followed easily by counting the number of paralyzed animals within the population (Cohen *et al.* 2006). Among many other related disease models, transgenic worms expressing fluorescently tagged polyQ stretches of different lengths in muscle cells (Morley *et al.* 2002), TDP-43 (Zhang *et al.* 2011), or ataxin-3 in neurons (Teixeira-Castro *et al.* 2011) have also been extremely useful in the study of proteostasis from an organismal perspective. Overviews of available disease reporter strains and methods for measurement of proteotoxicity-related phenotypes are described in detail elsewhere (Li and Le 2013; Volovik *et al.* 2014a).

The described protein aggregation models were successfully studied to characterize PNs important for protein aggregate handling and to find drugs that can mitigate the symptoms of disease-relevant proteinopathies. In particular, polyQ-expressing worms have been used in several unbiased genome-wide screens, which identified regulatory factors and pathways modulating the aggregation of aggregation-prone polyQ stretches expressed in muscle cells. The identified modifiers of polyQ aggregation are involved in RNA synthesis and processing, the initiation and elongation of translation, and protein degradation, as well as in protein folding (Nollen *et al.* 2004). These insightful genetic approaches nicely demonstrate the complexity of PNs, emphasizing the beneficial impact of well-balanced coordination between protein synthesis, folding, and degradation. *C. elegans* proteostasis models have also proven to be useful for chemical drug screening (Alavez *et al.* 2011; Calamini *et al.* 2011; El-Ami *et al.* 2014). In fact, a novel class of neuroleptics that is beneficial for ALS patients was identified using transgenic worms that express an aggregation-prone mutant form of TAR DNA-binding protein 43 (TDP-43) specifically in neurons (Patten *et al.* 2017).

## Organismal Regulation of Stress Response and Proteostasis

**Figure 3:**
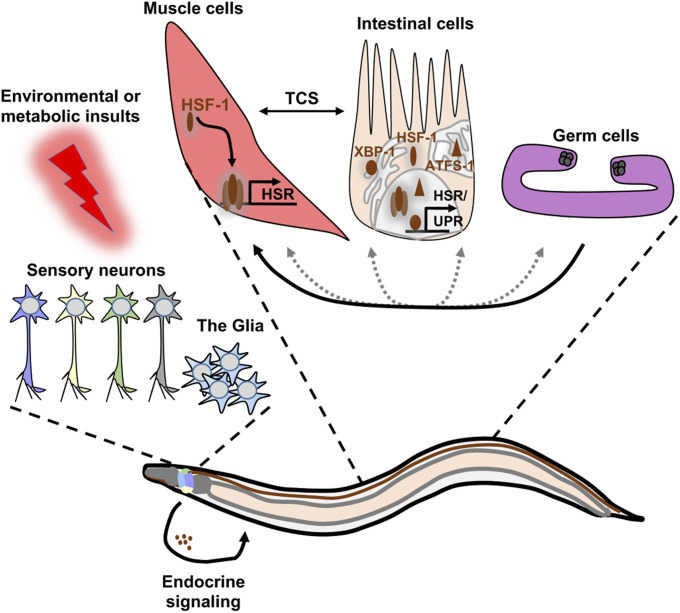
Organismal coordination of proteostasis networks. Schematic illustration of the organismal cross talk between cellular stress-response pathways in *C. elegans*. Perturbation of proteostasis in distinct cellular compartments results in cell-autonomous transcriptional responses termed the heat-shock response (HSR), the ER unfolded protein response (UPR), and the mitochondrial UPR, which mediate adaptive regulation of chaperones and proteolytic degradation pathways. Induction of these inducible gene expression programs is defined by the transcription factors HSF-1, XBP-1, and ATFS-1, which bind to specific regulatory elements on the DNA. Importantly, cellular responses can also be communicated to distal tissues by endocrine signaling, which integrates sensory perception of environmental insults, governed by serotonergic (blue), thermosensory (yellow), chemosensory (green), and additional neurons (gray). The Glial cells (light blue) are also involved in the orchestration of proteostasis. Muscle and intestinal cells also communicate by transcellular chaperone signaling (TCS), which coordinates chaperone activity across tissues. Finally, germ cells signal to other tissues to promote stress resistance and proteostasis. Thus, distal tissues are prepared to mitigate proteotoxic conditions that challenge the entire organism. Dashed gray lines indicate putative cross-communication between muscle (red), intestine (ocher), and the germ line (violet).

The ability of unicellular organisms and of cultured cells to induce the HSR (Lindquist 1986), as well as the apparent intracellular nature of the UPR^ER^ and UPR^mt^, supported the idea that these signaling pathways act cell-autonomously. However, this concept has been challenged by pioneering studies in *C. elegans* indicating that the activation of stress-response programs and proteostasis is coordinated organism-wide by the nervous system (Figure 3). The fundamental role of thermosensory neurons in heat sensing (Mori and Ohshima 1995) raised the question of whether these cells are involved in HSR regulation in somatic tissues. To address this idea, the Morimoto laboratory followed the expression of GFP by the inducible *hsp-70* promoter in *gcy-8* mutant worms. *gcy-8* encodes a receptor-type guanylyl cyclase that is critical for thermosensation by AFD neurons (Inada *et al.* 2006). Interestingly, AFD inactivation uncoupled the induction of the HSR in nonneuronal tissues as judged by reduction of green fluorescence and by decreased survival of heat-exposed *gcy-8*-mutants. Similarly, inactivating the AIY interneurons, which closely communicate with AFD thermosensory neurons, prevented HSR in distal tissues of heat-stressed worms (Prahlad *et al.* 2008). This seminal study indicated that AFD and AIY form a neuronal circuit that orchestrates the activation of the HSR across the organism, which raised interest in the underlying signaling mechanism. In fact, optogenetic stimulation of the AFD thermosensory neurons could demonstrate that HSF-1 is activated in peripheral tissues via serotonin release, even when the worms are cultured at the permissive temperature (Tatum *et al.* 2015). Since molecular chaperones promote protection from both heat shock and proteotoxicity (Brehme *et al.* 2014), it was thought that reducing the ability of the worm to respond to elevated temperatures would result in elevated sensitivity to proteotoxic stress. Nevertheless, abolishing the worm’s ability to induce the HSR by the inactivation of thermosensory (Prahlad and Morimoto 2011) or chemosensory neurons (Maman *et al.* 2013; Volovik *et al.* 2014b) was unexpectedly found to protect muscles from Aβ-mediated proteotoxicity. This phenomenon may be explained by a negative neuronal signal that averts cells from activating the HSR when the organism is exposed to the ambient temperature. Accordingly, the inactivation of these neurons releases distal cells from their negative regulatory mechanism and allows them to mildly activate the expression of molecular chaperones when proteotoxicity challenges the cell (Prahlad and Morimoto 2011). An additional indication that neurons orchestrate proteostasis in an organismal fashion was provided by the finding that knocking down caveolin-1 (*cav-1*), a gene that is expressed in the neurons of the adult nematode and is critical for the formation of specific membrane lipid microdomains, suppresses the rate of proteotoxicity caused by Aβ or polyQ aggregation in body-wall muscle cells. Surprisingly, the knockdown of *cav-1* had no effect on heat-stress resistance, further supporting the impression that the abilities to cope with heat and proteostasis are not necessarily linked (Roitenberg *et al.* 2018).

Nematodes that exhibit RNA interference (RNAi) hypersensitivity in neurons (Calixto *et al.* 2010) were used to identify additional regulators important for neuron-to-soma communication underlying the HSR. The knockdown of the G protein-coupled receptor (GPCR)-encoding gene *gtr-1* largely abolished HSR induction in nonneuronal tissues upon heat exposure. In contrast to *gcy-8*, *gtr-1* is mainly expressed in chemosensory neurons and not AFD, indicating that chemosensation is also involved in modulating proteostasis (Maman *et al.* 2013). Similarly, the worm’s innate immune response is also linked to neuronal signaling as the knockdown of the neuronal GPCR *octr-1* extends the survival of worms fed with the pathogenic bacterial strain *Pseudomonas aeruginosa*, by activating a noncanonical UPR^ER^ mechanism in the intestine (Sun *et al.* 2011). The surprising discovery that this UPR^ER^ mechanism is controlled by sensory neurons suggested that other UPR mechanisms might also be controlled cell-nonautonomously. To address this question and test whether neurons regulate organismal life span and stress resistance by activating the UPR^ER^, a constitutively active form of the transcription factor XBP-1 (XBP-1s) was expressed in different cell types. Intriguingly, expression of XBP-1s in the nervous system activates the UPR^ER^ in the intestine and muscles, extends life span, and triggers stress resistance (Taylor and Dillin 2013). These findings indicate that neurons communicate with remote tissues to regulate UPR^ER^ activity. To identify the molecular components that promote the signaling mechanism, nematodes that express XBP-1s in their neurons were employed for unbiased screening approaches. It turned out that lipid metabolism in general, and oleic acid in particular, are central molecules of the intertissue communication regulating UPR^ER^ (Imanikia *et al.* 2019b). Likewise, neuronal XBP-1s activates intestinal lysosomes to extend life span and confer enhanced proteostasis in worms expressing various proteotoxic proteins in different tissues (Imanikia *et al.* 2019a). The fundamental impact of UPR^ER^ as a key regulator of proteostasis is further supported by the discovery that expression of XBP-1s in neurons mitigates toxic phenotypes associated with protein aggregation of the tauopathy model. The stimulation of proteostasis triggered by neuronal XBP-1s depends on the ATF-6 branch of the UPR^ER^ (Waldherr *et al.* 2019). Intriguingly, a recent study could even show that exclusive expression of XBP-1s in only four astrocyte-like glial cells, namely the cephalic sheath glia cells of *C. elegans*, is sufficient for the cell nonautonomous activation of the UPR^ER^ in distal tissues. The underlying neuroendocrine signaling promotes proteostasis and resistance toward ER stress (Frakes *et al.* 2020).

Neurons were also found to govern and coordinate UPR^mt^ activity. Reducing the activity of the mitochondrial electron transport chain (ETC), by mutation (Feng *et al.* 2001) or RNAi (Dillin *et al.* 2002) activates the UPR^mt^. To examine in which tissue ETC activity regulates life span and to test whether neurons are involved in the regulation of UPR^mt^, the Dillin laboratory created worm strains expressing tissue-specific RNAi toward *cco-1*, a gene that encodes a component of complex IV of the ETC (Dillin *et al.* 2002). Knocking down *cco-1* exclusively in neurons was found to activate the UPR^mt^ in the intestine, indicating that similarly to the UPR^ER^, neuronal UPR^mt^ activates this stress-response program in distal tissues (Durieux *et al.* 2011). UPR^mt^-associated signaling regulates life span and proteostasis by epigenetic regulation (Merkwirth *et al.* 2016; Tian *et al.* 2016), and coordinates these functions with other stress-response pathways through lipid metabolism (Kim *et al.* 2016).

In contrast to stress-induced regulation of protein folding systems, little is known about adaptive changes of PNs caused by sensory perception. Use of GFP-tagged model substrates that allow the monitoring of ubiquitin-dependent protein degradation in *C. elegans* identified that the smell of different bacterial food sources modulates organismal proteostasis and longevity (Segref *et al.* 2011, 2014). In this context, the primary AWC olfactory neurons are central in sensing and transducing food-derived information, regulating ubiquitin fusion degradation and ERAD in intestinal cells. This neuron-to-gut communication is governed by the microRNA miR-71, which controls the level of the Toll-receptor-domain protein TIR-1 in AWC neurons. Thus, disruption of miR-71–tir-1 or loss of AWC olfactory neurons eliminates the influence of food source on chemotactic behavior and proteostasis (Finger *et al.* 2019). Showing that a neuronal olfactory circuit rewires proteolytic networks in intestinal cells, these findings provide a new concept for the regulation of food adaption, which is relevant to obesity and neurodegenerative diseases.

The organismal stress response is not only controlled by neuronal signaling. Increased proteotoxic stress in body-wall muscle cells of *C. elegans* was shown to upregulate the molecular chaperone HSP90 not only in muscle, but also in intestinal cells or neurons. This systemic stress response coordinates HSP90 expression by transcriptional feedback between different somatic tissues (van Oosten-Hawle *et al.* 2013). This transcellular chaperone signaling (TCS) pathway is differentially regulated by the transcription factor PQM-1. Upon neuronal induction, PQM-1 orchestrates TCS via the transmembrane protein CLEC-41, whereas PQM-1 involves the aspartic protease ASP-12 when triggered in intestinal cells (O’Brien *et al.* 2018). The TCS pathway mediates “transcellular” *hsp-90* induction in the muscle and protects against muscle-expressed amyloid structures and protein misfolding; however, the mechanistic details and molecular players regulating TCS need to be further addressed.

Signaling, which originates from germ cells, was also found to orchestrate proteostasis in remote tissues. First, unlike wild-type worms, animals that carry mutations in *glp-1* or *glp-4*, and thus have no germ line stem cells (GSCs) when developed at 25°, efficiently induce the expression of chaperones, and exhibit elevated resistance to heat stress and to the expression of aggregation-prone proteins, during reproductive adulthood (Shemesh *et al.* 2013). The ablation of GSCs reprograms the worm’s transcriptome to enhance proteasome activity and promote proteostasis. This mechanism depends on lipid signaling that activates the transcription factor SKiNhead 1 (SKN-1) (Steinbaugh *et al.* 2015). In addition, DNA damage activates an innate immune response that in turn enhances UPS activity and proteostasis in other tissues (Ermolaeva *et al.* 2013). Finally, a recent study has indicated that developing embryos affect the proteostasis of the hermaphrodite worm carrying them (Sala *et al.* 2020). Together, these surprising observations indicate intertissue communication mechanisms that rewire organismal PNs neuron-independently (Figure 3).

Collectively, these observations have pioneered the field of proteostasis research and indicate the existence of evolutionarily conserved mechanisms orchestrating PNs across multicellular organisms (Figure 3). Meanwhile, similar cell-nonautonomous regulation has been identified in higher organisms as serotonergic signaling was found to suppress ataxin 3 aggregation and neurotoxicity in mouse models of MJD (Teixeira-Castro *et al.* 2015). Similarly, food perception activates hepatic mTOR and Xbp1 signaling to promote ER adaptation in mice (Brandt *et al.* 2018), suggesting that manipulation of the underlying pathways might help to tackle human proteinopathies.

## Outlook: Therapeutic Role of Proteostasis

The observation that neurodegenerative disorders manifest late in life has defined aging as the major risk factor for the development of these progressive diseases (Amaducci and Tesco 1994) and raised the question of whether it is linked to declined functionality of the PN. The initial discovery of longevity-related genetic pathways in *C. elegans*, including the insulin/IGF signaling (IIS) cascade (Kenyon *et al.* 1993; Holzenberger *et al.* 2003), was fundamental in addressing conserved mechanisms of aging regulation. Knocking down the activity of the IIS receptor DAF-2 hyperactivates a series of transcription factors including DAF-16, HSF-1, and SKN-1, which boost stress resistance and life span (Knight and Bass 2001; Hsu *et al.* 2003; Tullet *et al.* 2008). Conclusively, knocking down the activity of *daf-2* protects worms from the Aβ-associated paralysis phenotypes that are mediated by DAF-16 and HSF-1 (Cohen *et al.* 2006). Over the years, numerous studies in *C. elegans* have indicated that IIS reduction protects animals from the toxicity of additional aggregation-prone, neurodegeneration-causing proteins including extended polyQ tracts related to HD and MJD (Morley *et al.* 2002; Teixeira-Castro *et al.* 2011). DAF-16 (Morley *et al.* 2002), HSF-1 (Kumsta *et al.* 2017), and PQM-1 (O’Brien *et al.* 2018) are also needed for the worm to resist polyQ-mediated proteotoxicity. These results show that the IIS cascade and its downstream transcription factors play important roles in adaptation of the PN to sustain the toxic aggregation of polyQ, Aβ, and other neurodegeneration-causing proteins.

Although proteostasis collapse appears to be progressively linked to aging, it does not always affect longevity. For example, knockdown of the E3 ligase NHL-1 protects worms from Aβ toxicity; however, it has no effect on life span (Volovik *et al.* 2014b). Another study has unveiled that proteostasis and longevity compete through the quality control E3 ligase CHIP, which on one hand triggers the degradation of misfolded proteins and on the other hand regulates the stability of the insulin receptor in worms, flies, and human cells (Tawo *et al.* 2017). These insights based on *C. elegans* studies demonstrate that the accumulation of protein aggregates triggers the IIS cascade, which is central to the mechanistic link between proteotoxic stress and life expectancy, and indicates that a loss of proteostasis is an inherent aspect of aging (López-Otin *et al.* 2013).

Together, these observations indicate the fundamental importance of proteostasis maintenance, especially through later stages of life. Concomitant rewiring and responsive adaptation of different nodes of PNs could harness the mechanisms that protect young individuals from proteinopathies, and prevent, or at least delay, neurodegeneration in the elderly without extending life span. Thus, investigating the coordination of cell type-specific and organismal PNs bears promise for the development of new therapeutic treatments against neurodegenerative disorders.
